# Radiation-Induced Sarcoma of the Head and Neck Following Radiotherapy for Nasopharyngeal Carcinoma: A Single Institutional Experience and Literature Review

**DOI:** 10.3389/fonc.2020.526360

**Published:** 2021-01-21

**Authors:** Jianlin Lou, Lin Jiang, Xinshen Dai, Huanhuan Wang, Jia Yang, Liang Guo, Meiyu Fang, Shengye Wang

**Affiliations:** ^1^Department of Head and Neck Surgery, Institute of Cancer Research and Basic Medical Sciences of Chinese Academy of Sciences, Cancer Hospital of University of Chinese Academy of Sciences, Zhejiang Cancer Hospital, Hangzhou, China; ^2^Zhejiang Chinese Medical University, Hangzhou, China; ^3^Department of Medical Oncology, Institute of Cancer Research and Basic Medical Sciences of Chinese Academy of Sciences, Cancer Hospital of University of Chinese Academy of Sciences, Zhejiang Cancer Hospital, Hangzhou, China; ^4^Department of Radiotherapy, Institute of Cancer Research and Basic Medical Sciences of Chinese Academy of Sciences, Cancer Hospital of University of Chinese Academy of Sciences, Zhejiang Cancer Hospital, Hangzhou, China

**Keywords:** nasopharyngeal neoplasms, sarcoma, radioactivity, surgical procedures, operative, treatment outcome

## Abstract

**Background and Objective:**

Radiotherapy (RT) is the primary treatment option for nasopharyngeal carcinoma (NPC), but it is associated with radiation-induced sarcomas (RISs). This study aims to investigate clinicopathological features and head and neck RIS prognosis after NPC RT.

**Methods:**

The medical and radiological records of the NPC patients (n =14,074) referred to Zhejiang Cancer Hospital, Hang Zhou, China between January 1995 and December 2018 were retrospectively reviewed. Among them, 22 patients were determined to have RIS after RT for NPC. The clinicopathological data, diagnosis, treatment, and follow-up results of 22 patients with RIS were analyzed in this retrospective research. All 22 patients underwent surgery as the main treatment. The levels of Overall Survival (OS) were determined through the Log-rank test and Kaplan–Meier method.

**Results:**

Among these patients, 13 were males and nine females with the male/female ratio of 1.44:1. The age during the primary RT of NPC ranged from 25 to 61 years old (median age: 37 years old). Patients’ ages ranged from 33 to 73 years old (median age: 52.5 years old) when diagnosed with RIS. The latency period for development of the RIS was between 3 and 36 years (median: 8.5 years) after RT. In this cohort, R0 resection was achieved in 13 cases, R1 resection in five cases, and R2 resection in four cases. During the follow-up period ranged from 2 to 102 months (median 14 months), 15 patients had died of the disease. Kaplan–Meier method showed that the 2-year, 3-year, and the 5-year cumulative OS rate was 50.3, 43.2, and 14.4%, respectively. The median survival time was 34 months. Surgical resection with R0 resection achieves a significantly better prognosis (P = 0.012). Patients under the age of 37 years old at the time of initial RT had a relatively better prognosis (P = 0.035).

**Conclusions:**

Although the incidence of RIS after RT of NPC is generally low, the treatment of RIS is very difficult. The RISs are associated with poor overall prognosis. R0 resection can improve the prognosis thus it should be considered as the primary and optimal choice for the treatment of RIS.

## Introduction

Radiotherapy (RT), used as a definitive and adjunctive option, is the primary and standard option for the treatment of head and neck carcinoma. Recent improvements in RT modalities for different cancers have improved the survival rates and long-term overall survival of patients with head and neck cancers (1). Moreover, improvements in the efficacy of RT using different modalities such as radiosensitizing agents and technological advances on tumor contouring have improved the survival of patients with cancers (2–4). These improvements have drawn great research attention towards treatment-related morbidity. Secondary malignancies caused by radiation, especially radiation-induced sarcomas, are perhaps the most severe late side effects of RT (5). As the oncological findings are improved, the incidence of sarcomas after irradiation is estimated to be as high as 0.3% in those who have survived neck and head cancer for a long time. Nevertheless, it is also accompanied by significant morbidity and mortality (6). Secondary malignancies induced by radiation, especially radiation-induced sarcomas (RIS) are among the main consequences associated with RT. RIS of the head and neck is not prevalent (7). Despite the limited number of cases, there have been records of a large range of histological subtypes including malignant fibrous histiocytoma, chondrosarcoma, osteosarcoma, fibrosarcoma, spindle cell sarcoma, angiosarcoma, and malignant schwannoma (8–10).

The main treatment of nasopharyngeal carcinoma (NPC) is RT and a significant population is at the risk of developing RIS in regions such as southern China, where NPC is highly prevalent (11). Improved RT outcomes result in the improved long-term survival of patients, which subsequently might increase the frequency of RIS of the head and neck cancer (12). For most of the malignancies caused by radiation, there has been evidence of a dose–response association between tumor risk and radiation. The incidence of RIS can be raised by a total dose of 55Gy or higher (13, 14).

NPC is a highly malignant cancer, with pathologically poorly-differentiated or undifferentiated in the overwhelming majority of NPC cases (15, 16). The primary and optimal treatment is RT, with a high dose of radiation for curative intention. The radiation field of NPC ranges from cranial base to supraclavicular, including an extensive area of the head and neck. Recent improvements in the therapeutic efficacy of RT have improved the long-term overall survival of patients with NPS after RT. Moreover, applications of RT have been increased recently. Incorporating these two events, it is expected that radiation-induced sarcoma (RIS) to become a serious challenge in these patients that can not be ignored.

RIS in the head and neck is a long-term serious complication following RT for NPC. The diagnostic criteria for RIS were first defined by Cahan and Woodard (17) and then revised by Murray et al. (18), and these criteria are still used. These criteria include (1) the tumor arises in a field that has been previously irradiated, (2) the subsequent tumor differs histologically from the first tumor, (3) no history of the new tumor at the time of RT, and (4) the new tumor is developed after a latency period following RT. Although the arbitrary cut-off used to differentiate between RIS and sporadic sarcomas is 3–4 years after RT, the post-RT median period of latency has been reported 10–12 years (6, 19–21). In addition, a novel multicenter investigation revealed how the second tumor incidence period associated with RIS is dramatically reduced by chemotherapy interaction (20).

The frequency of RIS is very low, which makes it difficult to conduct systematic reviews. However, several systematic reviews have been published recently (22–25). It had been reported that the incidence of RIS is between 0.03 and 0.8% in the early-stage (26). RIS may be related to the dose of radiotherapy at the site of occurrence. The highest risk of RIS has been reported to be observed at sublethal cellular doses (21, 27). Today, the most popular forms of RT to treat NPC are volumetric modulated arc therapy (VMAT) and intensity-modulated radiation therapy (IMRT). Such techniques rely on the development of the three-dimensional conformal RT (3D-CRT). This makes it possible for the target tissue to be irradiated with higher doses, and at the same time decreases the dose delivered to areas near the lesion. From a practical standpoint, these approaches guarantee less acute toxicity than conventional RT, meanwhile increasing the spectrum of low-dose irradiation (28–31). Therefore, its incidence has increased during the last few years. Early diagnosis is complicated because of the relatively long latency period. For a patient with RT history, in the cases of pain, mass, or trismus in the irradiated area, RIS should be taken into consideration (32).

Although the overall morbidity of RIS is relatively low, its overall prognosis is poor. Diagnosis and treatment are also intractable. From a clinical perspective, early detection of RIS can be difficult because of the induration and fibrosis of the irradiated field (33). Moreover, the general characteristics of a new sarcoma and RIS make it difficult to determine a specific distinction between these two: in fact, RIS and osteoradionecrosis are similar in gender ratio, median age, tumor grade, and median tumor size (34). Since the characteristics of RIS on CT and/or MR are heterogeneous, radiological findings can be problematic. In general, RIS cannot be readily differentiated from the recurrence of primary cancer and/or second primary lesions. However, RIS should always be considered as the most likely complication in the presence of a large scale, rapidly developing, and extensively invasive, osseous destructive lesion with a strongly heterogeneous appearance, and substantial contrast enhancement (35).

Recently different systematic reviews and cohort studies have been conducted on the frequency and clinical features of RIS in the neck and head cancer after RT. The main findings of these studies can be summarized as follows (22, 24, 25, 32, 36–39).

RISs of the head and neck are a rare but lethal consequence of RT, even for advanced RT modalities. The average frequency of RIS was about 0.15%. The mean latency interval between RT and occurrence of RIS in head and neck cancer is about 11 years, and the most common site for RIS of the head and neck is the sinonasal region. The most common histology is osteosarcoma and then fibrosarcoma, and surgery is the most frequently used treatment option.

Despite the increased use of RT, RISs of the head and neck have not increased significantly during the past two decades. In conclusion, the current evidence shows that surgery is the most efficient therapeutic option; however, its outcome remains poor.

Patients with these lesions are less likely to receive further radiation treatment, although chemotherapy seems to have a little benefit (40). The extremely aggressive nature of RIS, unavailability, and lack of re-irradiation feasibility and its poor chemosensitivity result in surgery to be the most effective treatment to enhance patient survival. In patients with macroscopically complete resection, a major improvement in disease-specific and total survivals was observed relative to cases where only incomplete surgery or re-irradiation had been carried out. Nevertheless, it may not be possible to achieve resection with microscopically negative margins, owing to RT-related sequelae, the complexity of the lesion, and the peculiarity of the anatomy. Furthermore, fascial planes are not respected by RIS during their development, and they also need wider, atypical resections. Although the aggressiveness required to properly resect can endanger vital or essential structures, surgeons look for a balance between surgical quality and unbearable sequelae (36).

In this study, we aimed to review the current evidence on the incidence, clinicopathological features, and management of RIS in the head and neck following RT for NPC. We also report our institutional experience with the diagnosis and management of RIS in the head and neck following RT for NPC for 25 years. Twenty two cases of RIS were retrospectively reviewed to analyze the clinical features and prognosis.

## Methods and Materials

### Clinical Data

A retrospective clinical analysis of RIS was extracted from a total of 14,074 NPC patients in our hospital between January 1995 and December 2018. In the cohort of these NPC patients, 10,076 cases were male and 3,998 cases were female with a male/female ratio of 2.52:1. At the initial diagnosis of NPC, the median age was 51 years old (range 8–95 years).

We used the following criteria for the diagnosis of a RIS which are a modification of those originally described: 1) They include a history of radiation, 2) the subsequently formed sarcoma must have emerged within the field that has been previously irradiated, 3) there is no sarcoma before radiation therapy in the irradiated field, 4) all sarcomas must have been proved histologically, and 5) relatively long latency between irradiation and a secondary sarcoma (17, 18, 41). Following a thorough assessment of the medical records of 14,074 NPC patients, 22 cases were diagnosed with RIS and qualified for further assessments.

For all patients qualified for our study, comprehensive clinical examination and auxiliary examination including nasopharyngeal fiberscope, computed tomography (CT)/magnetic resonance imaging (MRI) scan of the nasopharynx and neck, chest CT or X-ray, ultrasound of neck and abdomen, and radionuclide bone scan had been done before treatment to clarify the lesion and exclude the possibility of relapse or metastasis. All experimental procedures of this study were approved by the local ethics committee of Cancer Hospital of University of Chinese Academy of Sciences, Zhejiang Cancer Hospital, Hangzhou, China, which were in accordance with the ethical standards and regulations of human studies of the Helsinki declaration (2014).

### Treatment

#### Initial Treatment of Nasopharyngeal Carcinoma

All patients received definitive external beam radiotherapy (EBRT) of primary disease and bilateral neck with curative intent in Zhejiang Cancer Hospital. Before the year 2003, the primary disease was irradiated with a Cobalt-60 (CO60) while bilateral neck with a megavoltage. From the year 2003, the primary disease was irradiated with an X-ray beam while bilateral neck with an X-ray beam plus electron beam. Since the year 2006, whole-course IMRT of X-ray beam had been used. In our cohort, the primary tumor site (nasopharynx) received RT of 64–80 Gy, the involved region of head and neck received RT of 60–70 Gy, and the prophylactic region of head and neck received RT of 50–55 Gy. Among 22 patients who met the criterion of RIS, eight cases had RT combined chemotherapy; two cases had RT combined targeted therapy; three cases happened after IMRT, and one case happened after re-irradiation for 110 Gy.

#### RIS Treatment

All the 22 patients underwent surgery, of the 20 patients underwent radical surgery as the main curative-intended treatment, while two patients underwent surgery with palliative intent. Among 22 patients, 16 cases had surgery only, two cases had surgery followed by RT, one case had RT plus surgery, and three cases had surgery followed by chemotherapy. The re-irradiation dose was 50–62 Gy.

### Follow-Up and Statistical Analysis

In order to evaluate recurrence, patients received follow-up visits at the hospital every 3 months. When they did not attend the hospital, they were followed up through a telephone call or letter. The cumulative risk from the date of diagnoses of RISs was estimated by using the Kaplan–Meier approach. According to the log-rank test, there were differences in cumulative risk between groups. All P-values reported are two-sided and the significance level is considered to be P <0.05.

## Results

### Clinical Data

Through severe sifting, 22 cases with RIS are qualified for our study. The incidence of RIS is 0.16% (22/14,074). Among them, 13 were males and nine were females so that the males-to-females ratio was 1.44:1. The age at the time of primary RT of NPC ranged from 25 to 61 years old (median 37 years old). Patients’ ages ranged from 33 to 73 years old (median 52.5 years) when diagnosed with RIS. The latency period for development of the RIS is between 3 and 36 years old (median 8.5 years) after irradiation. The TNM stage of NPC initial treatment and the mode/dose of radiotherapy are shown in **Table 1**.

**Table 1:** The clinical data and treatment result of 22 RIS patients.

PN	Gender	TNM stage	Radiotherapy mode/dose	CCRT	Age atRT	Age atRIS	Latency(years)	Location of RIS	Pathology subtypes	The treatment of RIS	Result of resection	Outcome
1	M	T1N1M0	CO60/70GY	N	27	33	6	NC/PS	FS	RT+S	R0	ANED, 102 months
2	F	T1N1M0	CO60/66GY	N	25	48	23	NP	FS	S	R0	DOD, 6 months
3	M	T2N1M1	IMRT /70GY	Y	44	50	6	NE	FS	S	R0	ANED, 10 months
4	M	T1N0M0	6MV-X /68GY	N	29	37	8	OP	FS	S	R1	DOD, 2 months
5	M	T2N2M0	6MV-X /70GY	Y	61	64	3	LA	FS	S	R0	DOD, 15 months
6	F	T3N1M0	IMRT /70GY	Y	45	51	6	NP	FS	S	R2	DOD, 13 months
7	M	T2N2M0	CO60 /74GY	Y	36	60	24	NE	FS	S+RT	R0	ANED, 23 months
8	M	T2N1M0	CO60 /70GY	N	31	38	7	MA	OS	S	R1	DOD, 13 months
9	F	T1N0M0	CO60 /70GY	N	40	50	10	MA	OS	S	R0	DOD, 38 months
10	M	T3N1M0	6MV-X /80GY	Y	58	62	4	NC/PS	OS	S+CT	R2	DOD, 2 months
11	M	T2N2M0	CO60/70GY	Y	44	66	22	NE	OS	S	R1	DOD, 14 months
12	M	T1N1M0	CO60 /70GY	N	29	65	36	NC/PS	OS	S	R0	DOD, 42 months
13	F	T2N1M0	CO60 /74GY	N	37	43	6	NC/PS	MFHC	S+RT	R2	DOD, 7 months
14	M	T1N1M0	CO60/70GY	Y	47	54	7	NE	MFHC	S+CT	R1	DOD, 34 months
15	F	T1N1M0	CO60 /70GY	N	49	73	24	NE	MFHC	S	R0	DOD, 8 months
16	F	T1N1M0	6MV-X /70GY	N	31	34	3	NE	MS	S	R0	ANED, 21 months
17	M	T1N1M0	CO60 /70GY	N	33	51	18	NE	MS	S	R0	ANED, 68 months
18	F	T1N1M0	CO60 /70GY	N	34	51	17	MD	MS	S	R0	AWD, 9 months
19	M	T1N1M0	CO60 /70GY	N	28	58	30	NC/PS	US	S	R1	DOD, 51 months
20	M	T1N2M0	IMRT /130GY	Y	57	65	8	MA	US	S+CT	R2	DOD, 2 months
21	F	T1N0M0	6MV-X /70GY	N	45	54	9	MD	AS	S	R0	ANED, 9 months
22	F	T1N1M0	CO60 /70GY	N	37	57	20	NP	CS	S	R0	DOD, 39 months

TNM stage according to the American Joint Committee of Cancer (AJCC) staging system (7th edition). PN, Patient number; M, Male; F, Female; CCRT, Concurrent Chemoradiotherapy; NC/PS, Nasal cavity and paranasal sinuses; NP, Nasopharynx; NE, Neck; OP, Oropharynx; LA, Larynx; MA, Maxilla; MD, Mandible; FS, Fibrosarcoma; OS, Osteosarcoma; MFHC, Malignant fibrous histiocytoma; MS, Myofibro blastic sarcoma; US, Undifferentiated sarcoma; AS, angiosarcoma; CS, carcinosarcoma; S, Surgery; S+CT, Surgery + Chemotherapy; RT+S, Radiotherapy + Surgery; S+RT, Surgery + Radiotherapy; DOD, Dead of disease; ANED, Alive with no evidence of disease; AWD, Alive with disease.

The location of RIS included neck (NE, 7/22), nasal cavity and paranasal sinuses (NC/PS, 5/22), a maxilla (MA, 3/22), nasopharynx (NP, 3/22), mandible (MD, 2/22), oropharynx (OP, 1/22) and larynx (LA, 1/22. Pathology subtypes included 31.82% (7/22) of fibrosarcoma (FS), 22.73% (5/22) of osteosarcoma (OS), 13.64% (3/22) of malignant fibrous histiocytoma (MFHC),13.64% (3/22) of myofibro blastic sarcoma (MS), 9.09% (2/22) of undifferentiated sarcoma (US), 4.545% (1/22) of angiosarcoma (AS) and 4.55% (1/22) of carcinosarcoma (CS) (**Table 1**).

**Figure 1:**
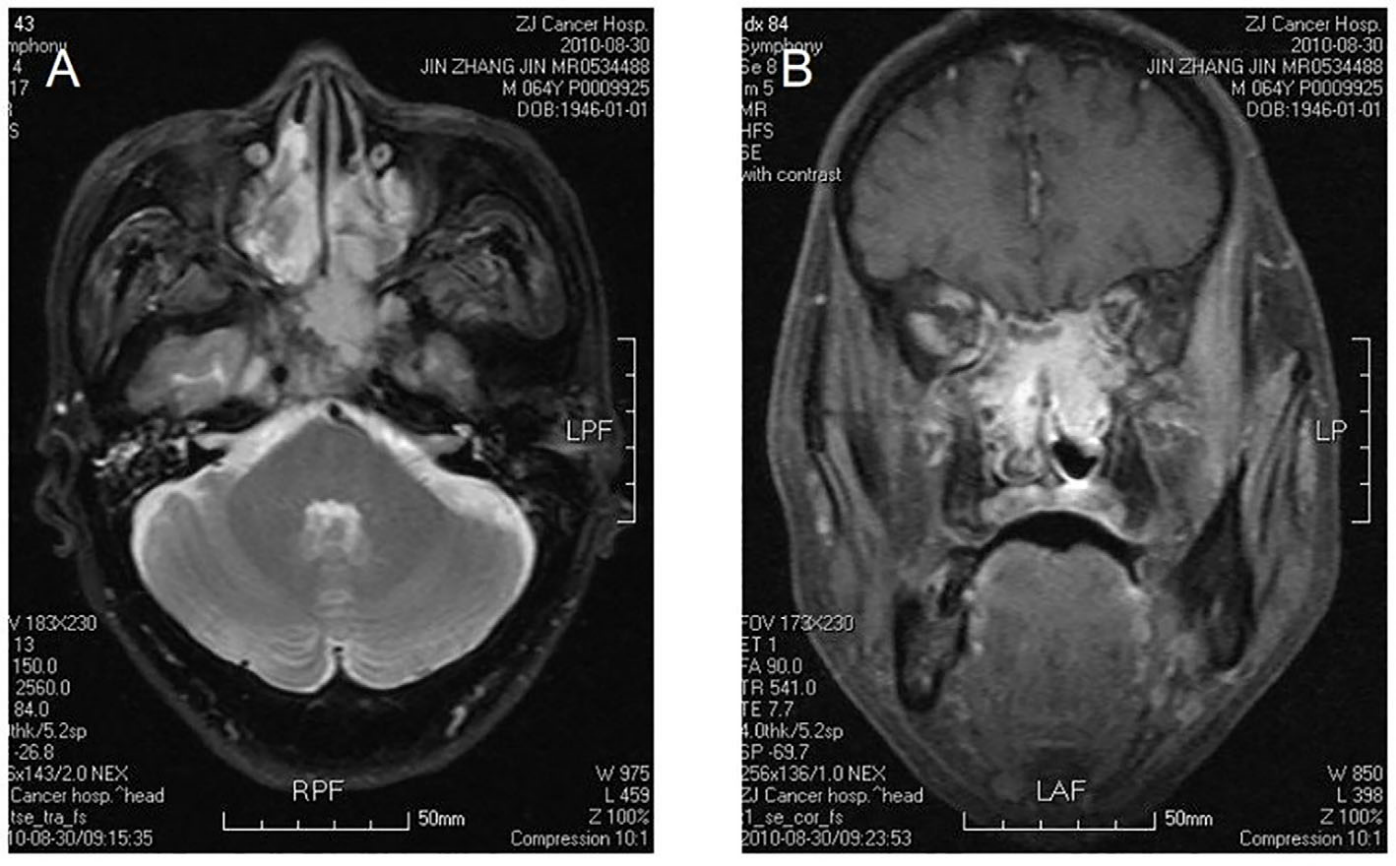
Bilateral nasal cavity and sinus osteosarcoma. MR image shows bilateral postirradiation osteosarcoma in a mixture of nasal and paranasal sinuses. In magnetic resonance imaging, the signal was isointensity in T1 and high in T2 weighted images in bilateral postnaris, ethmoidal cellules and sphenoid sinus. The lesion violates medial orbital wall and musculus rectus medialis of left eye with irregular morpha. There was evident intensification after contrast. The signal of skull bone is abnormal **(A, B)**.

**Figure 2:**
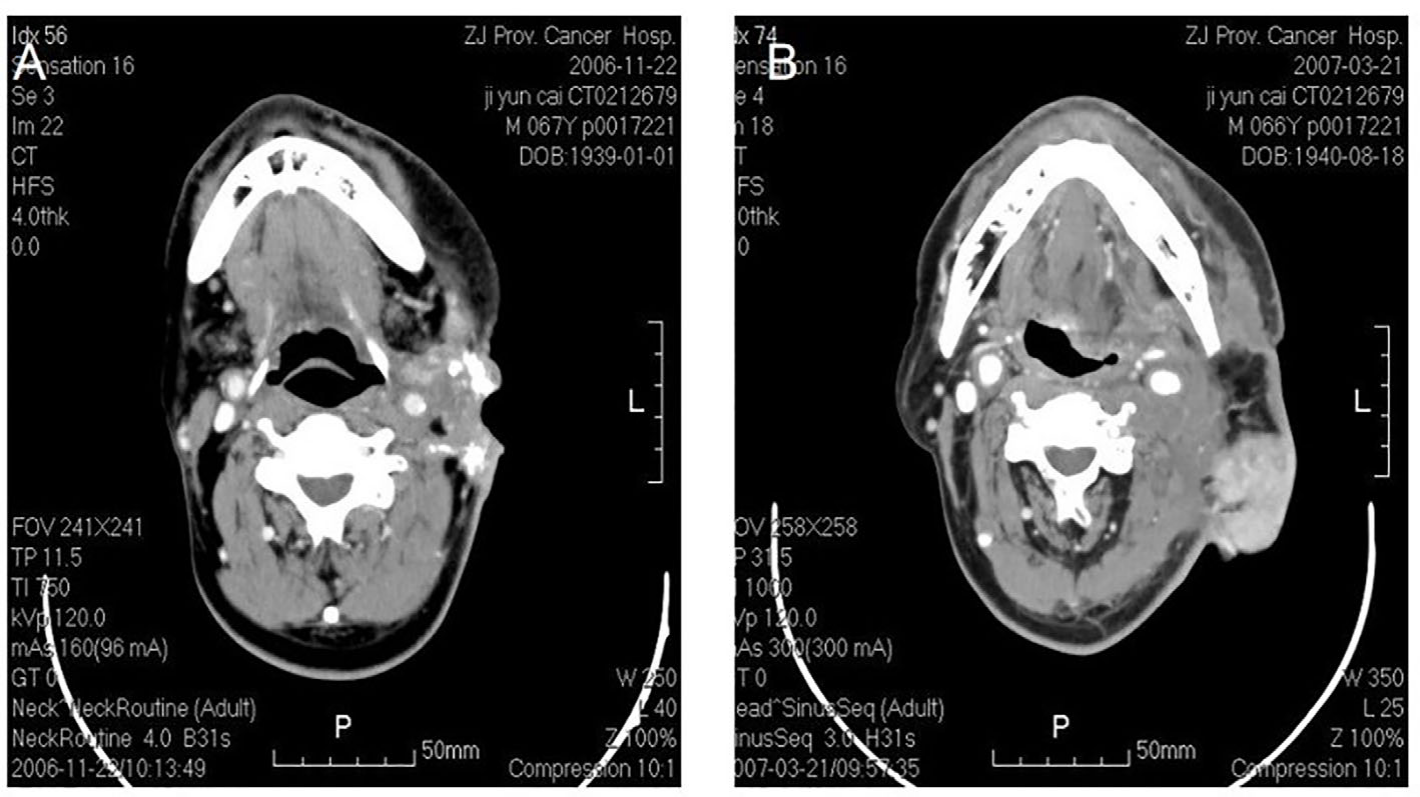
Osteosarcoma in left neck. CT shows an irregular soft tissue imaging in the left neck, with heterogeneited density, several schistose calcification in it. CT also shows that the lesion has been heterogeneited enhanced after contrast, with obscure margin to the left cervical vascular, with superficial elcosis. **(A)** The lesion recurred after 4 months. **(B)** The picture on the right shows pedicled pectoralis major myocutaneous flap with surrounded recurrent lesions.

**Figure 3:**
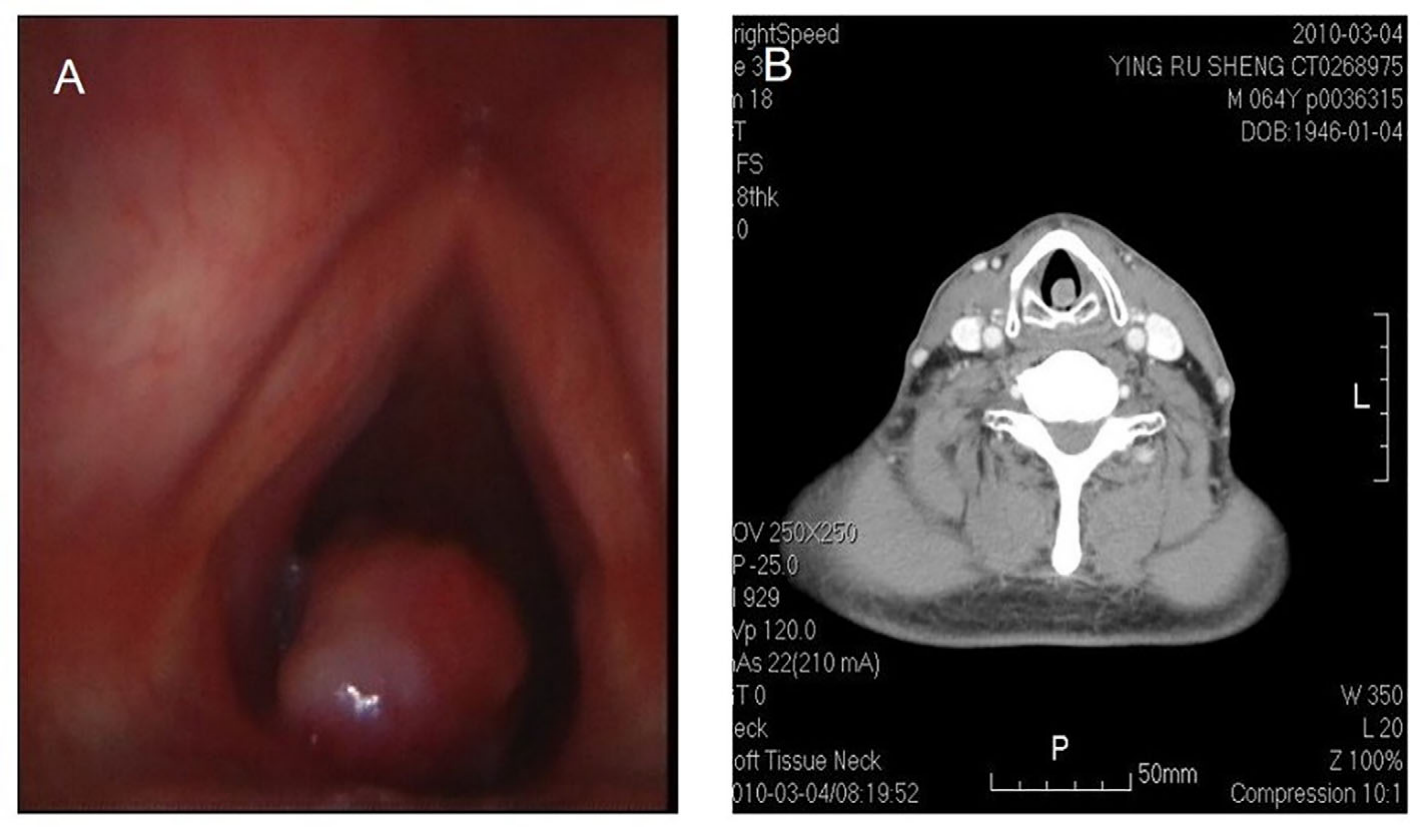
Fibrosarcoma in throat. Laryngoscope shows a nodulated neoplasm in the left posterior wall of hypolarynx, with surface relatively flattening **(A)**. CT also shows that CT a nodulated neoplasm in infraglottic portion, with medium isotropical enhancement after contrast. The lesion is connected with posterior wall **(B)**.

**Figure 4:**
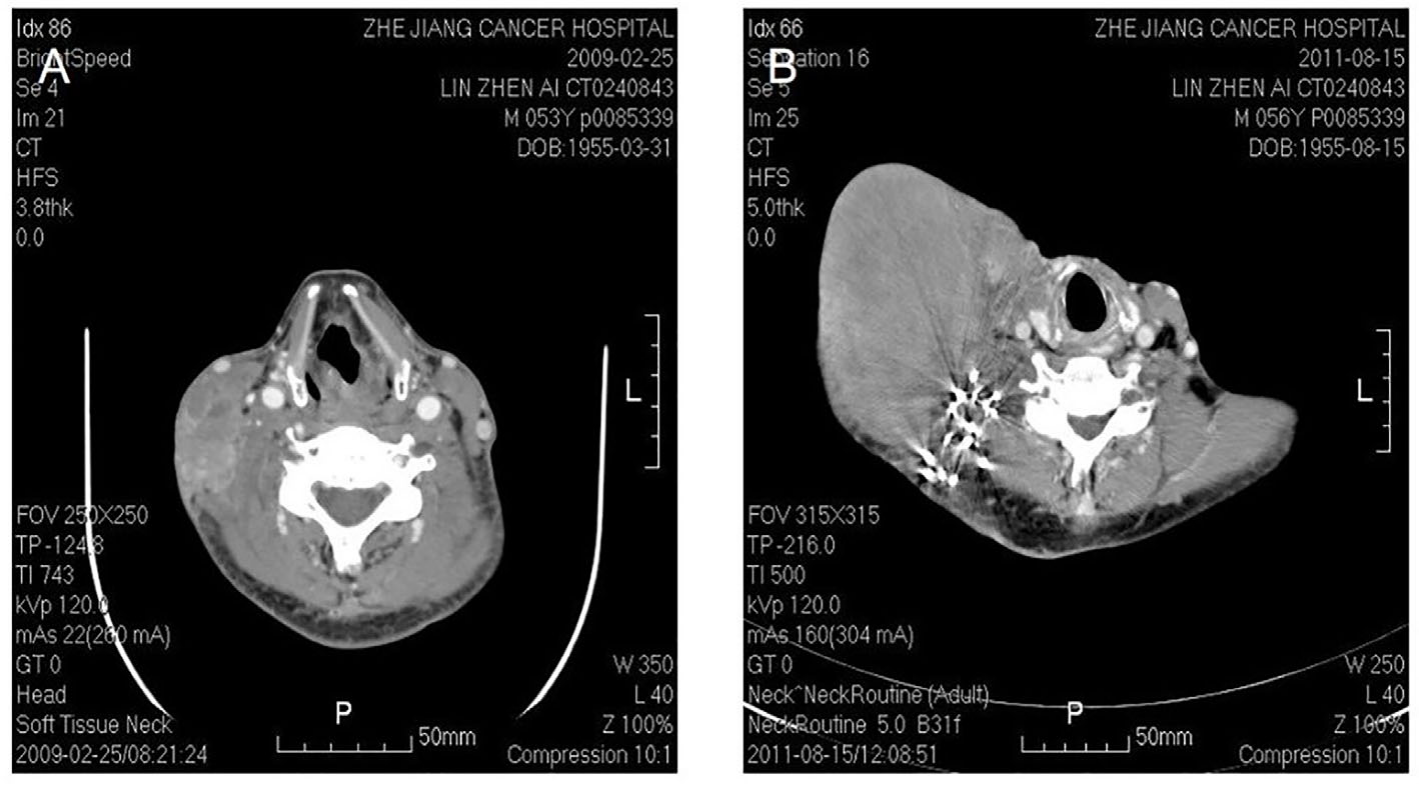
Malignant fibrous histocytoma in right neck. CT shows soft tissue image nearby sternocleidomastoideus in the middle to lower portion of right neck, with illegible margin and patchy enhancement after contrast. The lesion violates circumambient skin, subcutaneous tissue and sternocleidomastoideus **(A)**. The picture on the right shows that recurrent lesion with superficial elcosis is surrounding the right cervical vascular, treated by high density particle **(B)**.

Imaging manifestations of typical cases and pathological sections of typical cases are presented in **Figures 1**–**4**.

### Treatment Result

In our cohort, R0 resection was achieved in 13 cases, R1 resection in five cases, and R2 resection in four cases. In addition,10 patients had a tracheotomy, five cases had neck skin defect covered with pedicled pectoralis major myocutaneous flap, and two cases had mandible defect repair by free anterior lateral femoral flap and free fiber bone flap with titanium plate, respectively. Main complications after operation: two cases had bleeding, one case flap necrosis, and one case pulmonary infection after the operation.

### Survival Analysis and Prognostic Factor

**Figure 5:**
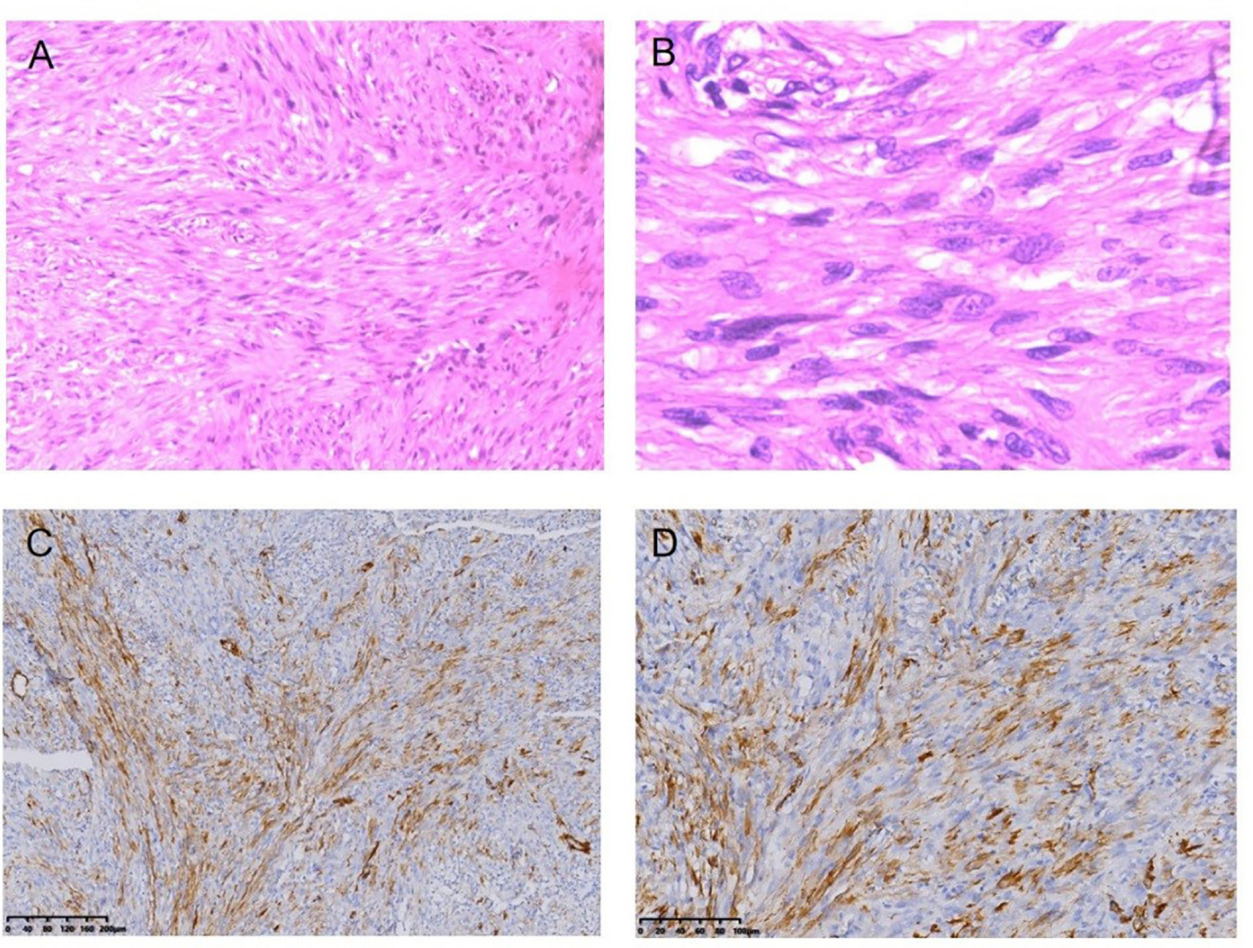
Fibrosarcoma following RT for NPC HE staining (*100) **(A)**, HE staining (*400) **(B)**, Immunohistochemical (IHC) staining: SMA + (*100) **(C)**, SMA + (*200) **(D)**.

**Figure 6:**
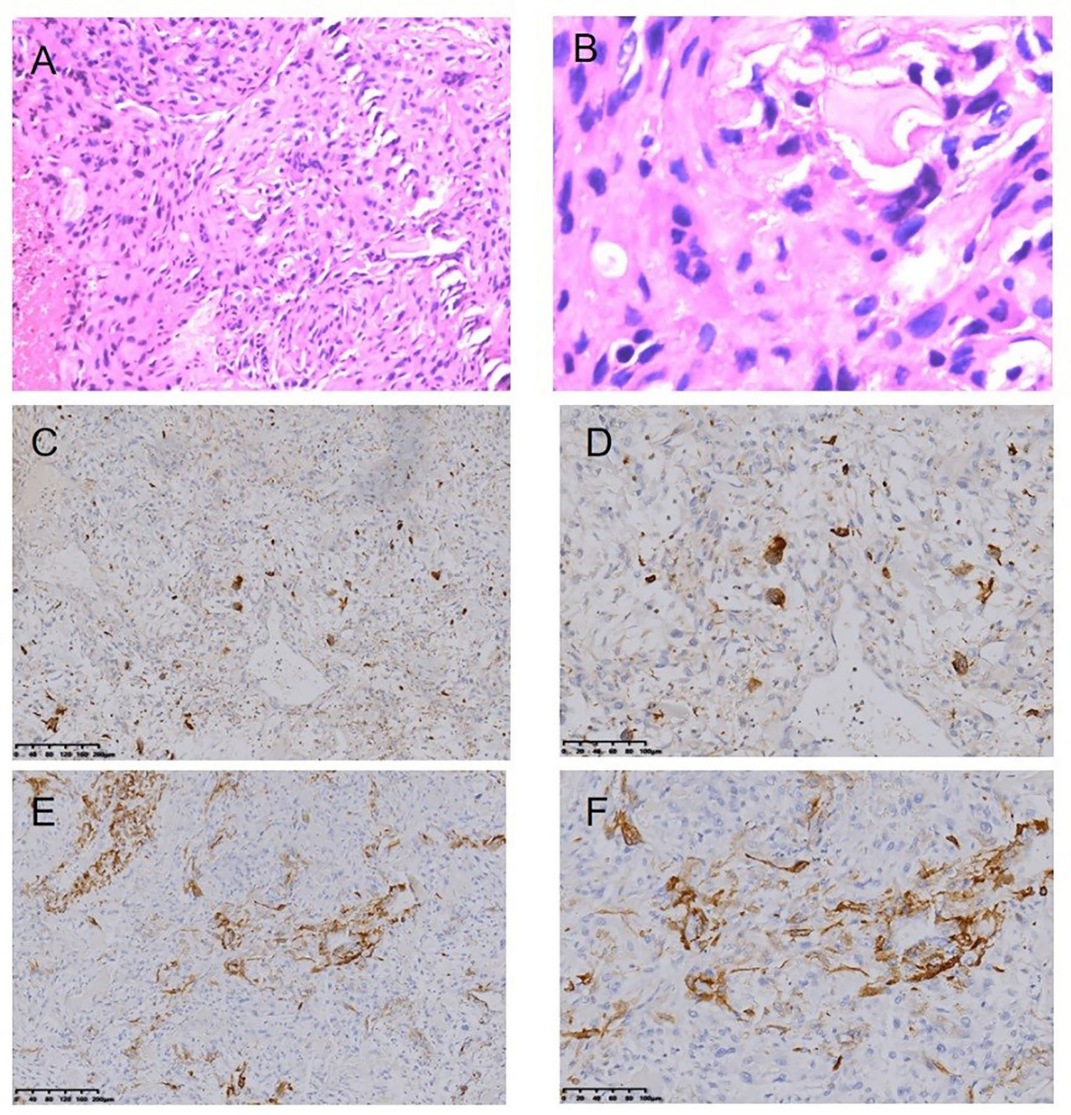
Osteosarcoma following RT for NPC HE staining (*100) **(A)**, HE staining (*400) **(B)**, Immunohistochemical (IHC) staining: CD68+ (*100) **(C)**, CD68+ (*200) **(D)**, SMA+ (*100), **(E)**, SMA+ (*200) **(F)**.

**Figure 7:**
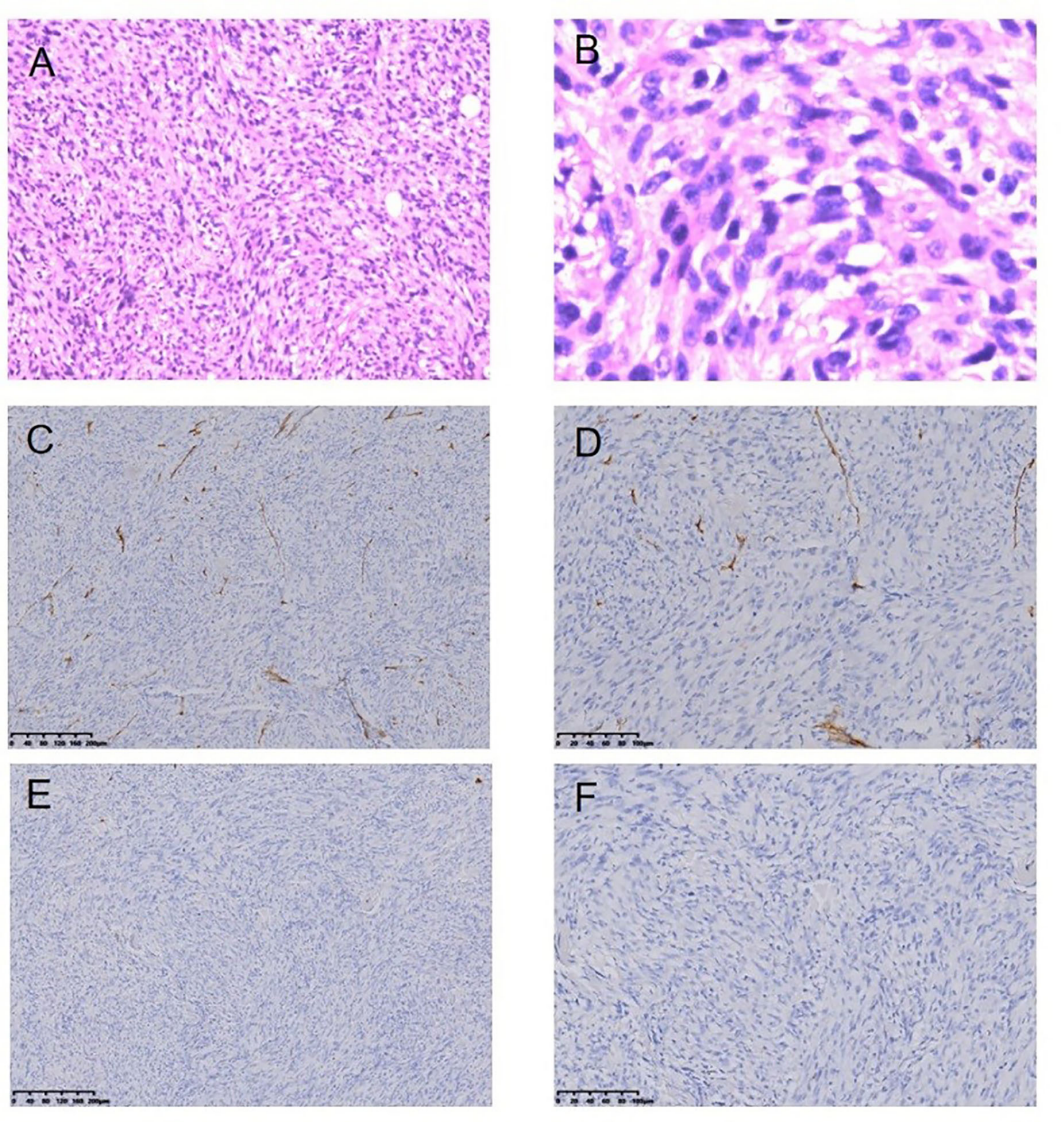
Malignant fibrous histiocytoma following RT for NPC HE staining (*100) **(A)**, HE staining (*400) **(B)**, Immunohistochemical (IHC) staining: CD34− (*100) **(C)**, CD34− (*200) **(D)**, SMA− (*100) **(E)**, SMA− (*200) **(F)**.

**Figure 8:**
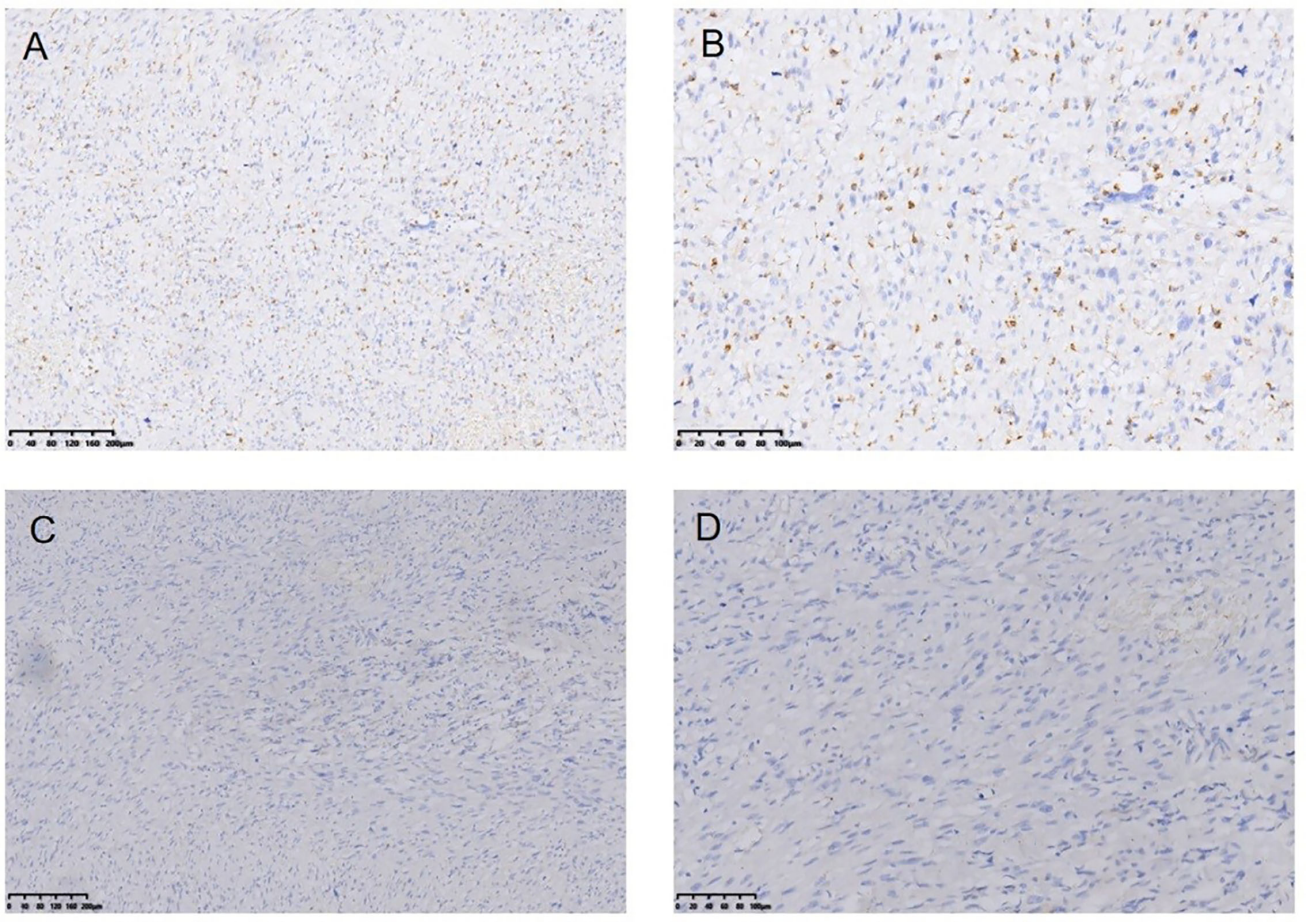
Myofibro blastic sarcoma following RT for NPC Immunohistochemical (IHC) staining: CD68− (*100) **(A)**, CD68− (*200) **(B)**, EMA− (*100) **(C)**, EMA-(*200) **(D)**.

**Figure 9:**
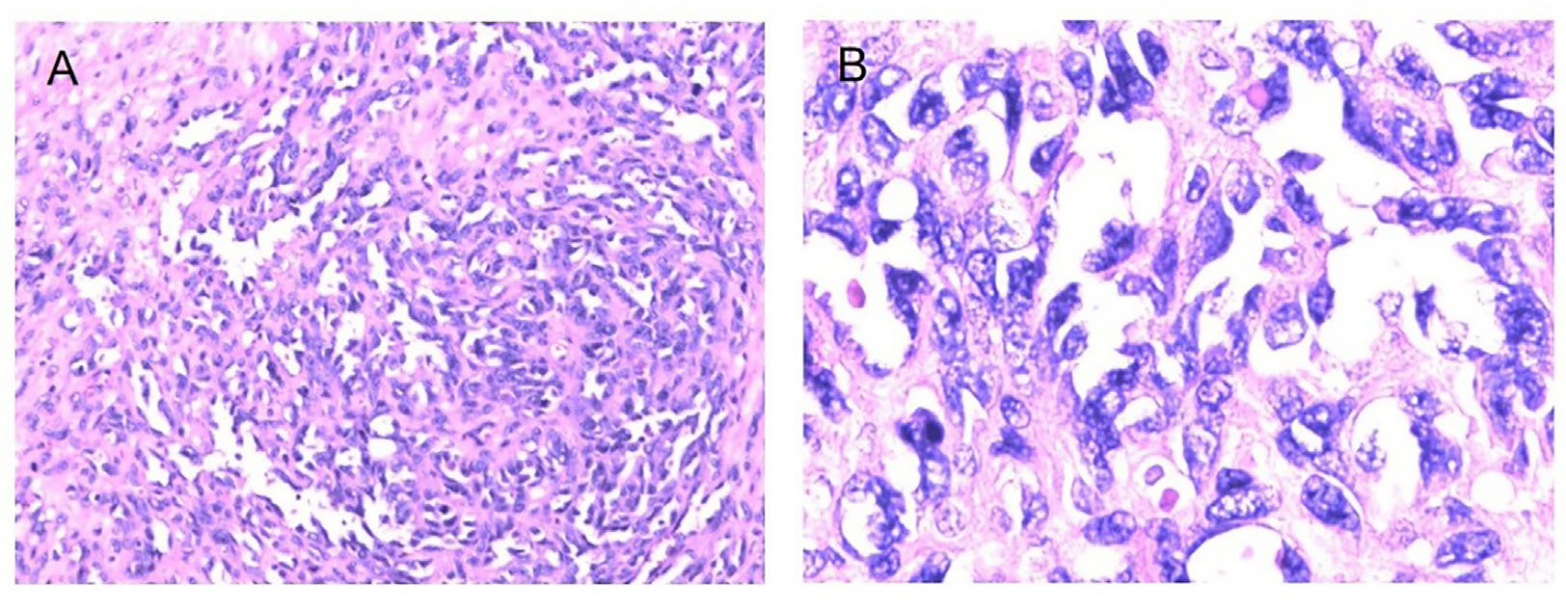
Angiosarcoma following RT for NPC HE staining (*100) **(A)**, HE staining (*400) **(B)**.

**Figure 10:**
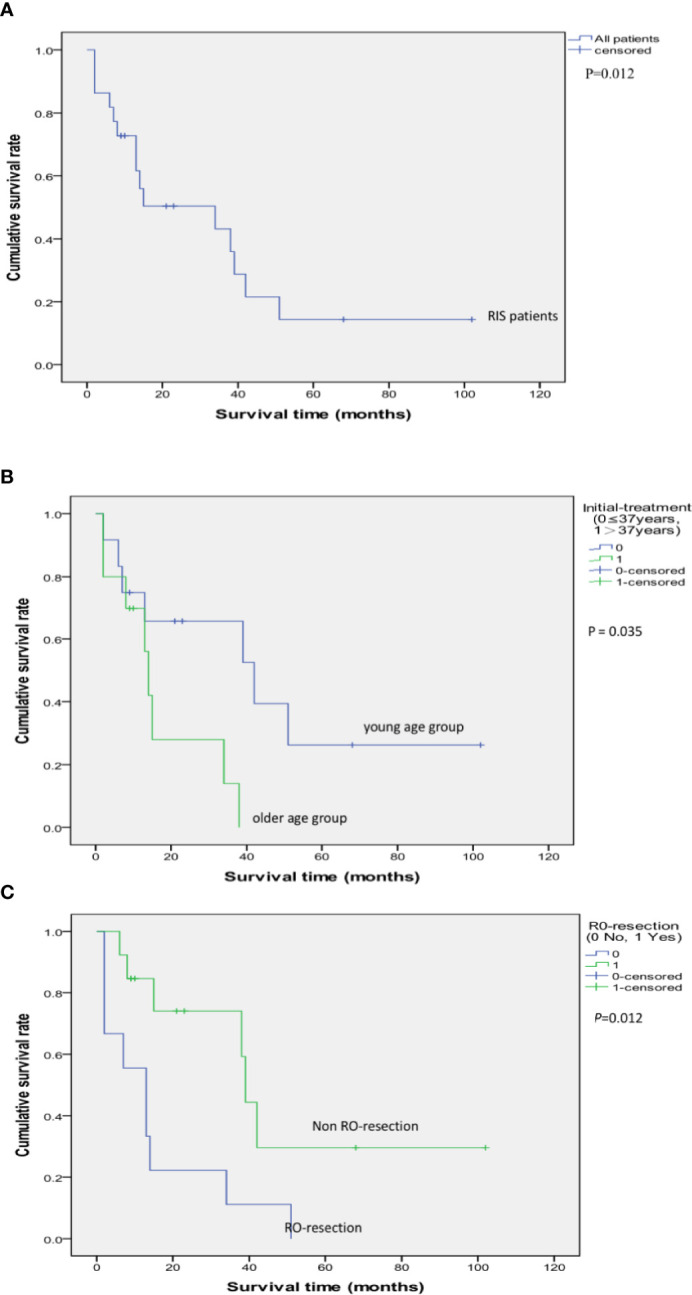
Kaplan–Meier Survival Curve of 22 cases of RIS following RT for NPC **(A)**; The impact of R0 resection to the survival rate of RIS patients (P = 0.012) **(B)**; The impact of age: Survival comparison between the young age group (initial treatment age of NPC ≤ 37 years old) and the older age group (initial treatment age of NPC > 37 years old) (P = 0.035) **(C)**.

Up to February 2019, during the follow-up period ranged from 2 to 102 months, 15 (68.18%) of them died of the disease. Among the 15 cases, six cases died within 1 year, four cases within 1–2 years. Kaplan–Meier methods resulted in that the 2-year, 3-year, and 5-year cumulative overall survival (OS) rate was 50.3, 43.2, and 14.4%, respectively. The median survival time was 34 months (**Figure 5**). Surgical resection with R0 resection achieves a significantly better prognosis (P = 0.012). Patients younger than 37 years of age had a relatively better prognosis at the time of initial RT (P = 0.035) (**Table 2**, **Figures 6**–**10**).

**Table 2:** The univariate analysis for the survival of RIS.

Clinical Factor	Number of cases	Overall Survival		χ^2^ value	P value
		2 years (%)	3 years (%)		
Gender					
Male	13	51.3	41.0		
Female	9	50.0	50.0	0.741	0.389
NPC initial age (yrs)					
≤37	12	65.6	65.6		
>37	10	28.0	14.0	4.433	0.035^*^
RIS initial age (yrs)					
≤52	11	48.5	48.5		
>52	11	51.9	39.0	0.390	0.532
Latency(months)					
≤8	11	31.8	15.9		
>8	11	70.1	70.1	1.628	0.202
RIS location					
Cervix	7	68.6	34.3		
Not Cervix	15	41.7	41.7	1.254	0.263
R0 resection					
Yes	13	74.0	74.0		
No	9	22.2	11.1	6.298	0.012^*^
Repair of skin flaps					
Yes	9	38.9%	0		
No	13	53.8%	53.8%	0.641	0.423
Combined treatment					
Yes	6	40.0%	40.0%		
No	16	52.4%	43.7%	0.004	0.950

## Discussion

RT causes RIS as a complication in the long-term, which becomes an important factor of overall survival for NPC patients. RIS is rare, which makes it difficult to study systematically (19, 41). It had been reported that the incidence of RIS is between 0.03 and 0.8% in the early-stage (26). Wei et al. have reported that the cumulative incidence of RIS is 0.06–0.17% in the head and neck (19). Yang et al. retrospectively analyzed 27,714 NPC patients from December 1998 to September 2012, and a total of 69 patients (0.25%) were found who had RIS in the head and neck after RT for NPC (23). In our study, 22 cases of RIS were sifted from a total of 14,074 NPC patients in our hospital within 24 years. The incidence of RIS is 0.16% (22/14074), which is similar to other reports.

The mechanism of RIS is not yet clear. A recent study showed that the incidence and interval of RIS are affected by the following factors: hereditary susceptibility, the type of primary tumor, age of initiative irradiation, the dose and area of irradiation, the toleration of the irradiated tissues, and combination of chemotherapy (23, 42). Besides, a novel multicenter investigation revealed how the second tumor incidence period associated with RI is dramatically reduced by chemotherapy interaction (20). In our study, the ratio of males to females with NPC was 2.52:1. The median age at the initial treatment in the huge NPC cohort is 50 years old. While the ratio of males to females with RIS was 1.44:1. In the RIS cohort, the median age during the primary RT of NPC was 37 years old (52.5 years old when diagnosed RIS). In our series, middle-aged NPC women are probably more susceptible to RIS after RT. Because of the research time limit and other reasons, this study did not find that IMRT and concurrent radiotherapy and chemotherapy may increase RIS or shorten the interval time.

RIS has a long latency. Early detection and treatment is the key point. So it is quite important to make an early correct diagnosis. The criterion of RIS is relatively definitive while some modifications have been suggested. Since the intervals between the NPC radiation and the occurrence of RIS are long, early symptoms are easy to be neglected or hidden behind. It is not so easy to make a quick and correct diagnosis clinically because of the morphologic change after radiation and inference of tumor recurrence itself. Until the clinical emergence of RIS, a fairly long asymptomatic duration would have passed after irradiation. Although being controversial, it has been longer than the 3 to the 5-year-cure period (33, 35). In our series, the interval between NPC and RIS are 3 years and 36 years (median 8.5 years). Therefore, regular follow-up is necessary for long survivals after RT for NPC, especially for initially early-stage NPC patients. When a neoplasm arises in the area within the radiotherapeutic beam, we should be alert of recurrence as well as the possibility of RIS. Only by early diagnosis and in-time treatment, the prognosis could be improved. RIS is always found advanced stage by detection of clinical symptoms. During the follow-up period, the main emphasis is paid to the radiology strategies such as CT or MRI. Since the varieties of pathologic types and imaging manifestations, it is difficult to differentiate the early RIS from irradiation alteration. So we should attach importance to the master of the differences (33, 35).

Due to the extremely aggressive nature of RIS and its high resistance against chemotherapy, the most critical prognostic factor and the only currently available curative method is radical surgery (22). Because of the invasive feature and advanced stage of RIS after RT in the head and neck, it is difficult to operate radically based on unsacrificing important organs. Especially when a tumor infiltrates into the skull base or internal carotid artery, dissection of vital organs may cause serious complications. Xi et al. reported the data of 49 cases who received treatment among 53 cases of diagnosed RIS. Xi et al. reported that the rate of the 3-year overall survival was 32.4% in 49 treated patients among totally 53 patients with RISs, and the median survival was 21.2 months. The only major prognostic factor for survival was complete surgical resection (41). Chan et al. reported that twenty of 25 patients of RISs received surgery with curative intent, but 14 of them ultimately had microscopic positive margins, including six cases of positive margin in the frozen-section examination and eight cases in the paraffin-section examination. All these 14 patients underwent postoperative adjuvant chemoradiation, and six of them also received brachytherapy. These postoperative adjuvant therapies seem to improve locoregional control. The 5-year overall survival rate is 24.2% and the median overall survival time is 2.21 years. Surgical resection with clear margins provides dramatically improved survival. In patients with an aggressive form of the disease, surgery also helps to relieve pain, bleeding, and trismus, even though not achieving the radical goal (43). Wei et al. reported that the rate of 3-year overall RIS survival was 19.1%, and the 3-year survival rate with no disease was 11.1% (19).

The overall prognosis of RIS was poor. In our cohort, the 2-, 3-, and the 5-year cumulative OS rate was 50.3, 43.2, and 14.4%, respectively. Twenty-two patients all underwent surgery. R0 resection was achieved in 13 cases. R0 resection achieves a significantly better prognosis (P = 0.012). RIS in NPC can be considered as a strongly malignant disease with a low prognosis due to restricted operability and lack of effective and sometimes tolerant adjuvant treatment. Radical surgery may improve prognosis. We need more future research as to comprehensive treatment. Yang et al. report the first clinical use of carbon ion radiotherapy (CIRT) for rescue therapy of locally recurrent (LR) or RT-induced secondary HNS following surgery and/or RT. The study revealed that salvage CIRT is capable of effectively controlling the tumor in the short term with few observed acute and late toxicities. Further research is needed to verify the effectiveness and safety of salvage CIRT in this group of patients (44). In addition, some scholars believe that neoadjuvant therapy with re-irradiation even without chemotherapy can be beneficial for RIS treatment. In such a clinical setting, this, in fact, gives rise to an overwhelming need for a systematic multidisciplinary approach to search for adjunctive techniques to treat a very gloomy disease (45).

In this study, the results showed that young NPC patients were prone to RIS, and patients younger than 37 years old during initial RT for NPC would have a better prognosis after RIS treatment (P = 0.035). Therefore, we can draw a conclusion that the long-term survival rate after treatment is high, so physicians need to be especially alert to the occurrence of RIS. And once found, they need to fight for opportunities and take radical treatment.

Despite the small sample size of our current study, our study gives proof of concept treatments of RIS. In our study, seven cases (31.82%) had RISs in the site of neck, while Cai et al. reported that 10 out of 59 cases (16.9%) had RISs in the site of neck (35). It is indicated that the irradiated region of neck was liable to RISs. Clinically, the lesion of RIS in neck was relatively easy to be detected early and the radical resection rate is relatively high, which may have a better prognosis. But in this study, there was no statistically significant difference in this group. The sample size needs to be further expanded. Despite the limitations, this study reported that although the incidence of RIS after RT of NPC is generally low, the treatment of RIS is very difficult. The RISs are associated with poor overall prognosis. R0 resection can improve the prognosis thus it should be considered as the primary and optimal choice for the treatment of RIS. In contrast to previous studies, the current study provides a detailed and larger timespan data of 20 years, giving a summary of the last two decades for RIS prognosis and treatment. The current study will be greatly helpful for the physician and healthcare providers for prognosis and determining treatment modules for the RIS among the different populations. Furthermore, the current study will help the researchers to distill this concise information to advance the current researchers direction.

## Conclusion

Our findings show that although the incidence of RIS after RT for NPC was relatively low, the treatments of RIS were difficult and the prognoses were poor. Currently, surgery remains the optimal strategy for treating RISs. We should comprehensively evaluate the possibility of radical surgery and systematic treatment according to several factors such as tumor pathologic type, radiology data, patients’ status and previous radiation dose. For patients who have the chance to get radical dissection, we should positively operate them with possibly R0 resection, otherwise, multiple discipline treatment and clinical trials could be strongly recommended.

## Data Availability Statement

The original contributions presented in the study are included in the article/supplementary materials; further inquiries can be directed to the corresponding author.

## Ethics Statement

The studies involving human participants were reviewed and approved by the Medical Ethics Committee of Zhejiang Cancer Hospital. The patients/participants provided their written informed consent to participate in this study.

## Author Contributions

JL and SW conceptualized the study. LJ contributed to the methodology. XD provided the software. HW, JY, and LG validated the study. MF performed the formal analysis. LJ investigated the study. LG provided the resources. JL conducted the data curation. JL wrote and prepared the original draft. SW wrote, reviewed, and edited the manuscript. JL conducted the visualization. LG supervised the study. SW conducted the project administration. LG, JL, and MF acquired the funding. All authors contributed to the article and approved the submitted version.

## Funding

This study was supported by the Major Science and Technology Projects of Zhejiang Medical and Health Program (No: WKJ-ZJ-1712, 2015ZDA007) and the General Research Projects of Zhejiang Medical and Health Program (No: 2017KY029, 2021KY571), Zhejiang Administration of Traditional Chinese Medicine Foundation (No: 2018ZB025, 2015ZQ010).

## Conflict of Interest

The authors declare that the research was conducted in the absence of any commercial or financial relationships that could be construed as a potential conflict of interest.
